# *Pantoea stewartii* WceF is a glycan biofilm-modifying enzyme with a bacteriophage tailspike-like fold

**DOI:** 10.1016/j.jbc.2021.100286

**Published:** 2021-01-13

**Authors:** Tobias Irmscher, Yvette Roske, Igor Gayk, Valentin Dunsing, Salvatore Chiantia, Udo Heinemann, Stefanie Barbirz

**Affiliations:** 1 Physikalische Biochemie, Universität Potsdam, Potsdam, Germany; 2 Department Theory and Bio-Systems, Max Planck Institute of Colloids and Interfaces, Potsdam, Germany; 3 Crystallography, Max-Delbrück-Centrum für Molekulare Medizin, Berlin, Germany; 4 Physikalische Zellbiochemie, Universität Potsdam, Potsdam, Germany; 5 Institut für Chemie und Biochemie, Freie Universität, Berlin, Germany

**Keywords:** biofilm, *Pantoea stewartii*, bacterial pathogenesis, glycoside hydrolase, exopolysaccharide, oligosaccharide, X-ray crystallography, parallel beta-helix, bacteriophage tailspike, CE, capillary electrophoresis, EPS, exopolysaccharide, IMAC, immobilized metal affinity chromatography, MBTH, 3-methyl-2-benzothiazolinon-hydrazone, RU, repeat unit, TSP, tailspike protein

## Abstract

Pathogenic microorganisms often reside in glycan-based biofilms. Concentration and chain length distribution of these mostly anionic exopolysaccharides (EPS) determine the overall biophysical properties of a biofilm and result in a highly viscous environment. Bacterial communities regulate this biofilm state *via* intracellular small-molecule signaling to initiate EPS synthesis. Reorganization or degradation of this glycan matrix, however, requires the action of extracellular glycosidases. So far, these were mainly described for bacteriophages that must degrade biofilms for gaining access to host bacteria. The plant pathogen *Pantoea stewartii* (*P. stewartii*) encodes the protein WceF within its EPS synthesis cluster. WceF has homologs in various biofilm forming plant pathogens of the *Erwinia* family. In this work, we show that WceF is a glycosidase active on stewartan, the main *P. stewartii* EPS biofilm component. WceF has remarkable structural similarity with bacteriophage tailspike proteins (TSPs). Crystal structure analysis showed a native trimer of right-handed parallel β-helices. Despite its similar fold, WceF lacks the high stability found in bacteriophage TSPs. WceF is a stewartan hydrolase and produces oligosaccharides, corresponding to single stewartan repeat units. However, compared with a stewartan-specific glycan hydrolase of bacteriophage origin, WceF showed lectin-like autoagglutination with stewartan, resulting in notably slower EPS cleavage velocities. This emphasizes that the bacterial enzyme WceF has a role in *P. stewartii* biofilm glycan matrix reorganization clearly different from that of a bacteriophage exopolysaccharide depolymerase.

Many microorganisms produce extracellular matrices composed of polymeric substances to organize themselves in microbial communities (1, 2). The production of these biofilms exerts a multitude of effects not only on the lifestyle of the embedded cells, but also on their interactions with the environment and their pathogenicity. Biofilms specifically regulate the access of external substances such as antimicrobial drugs, functioning clearly beyond simple diffusion barriers (3). Rather, biofilms show high spatiotemporal dynamics due to complex and multidimensional regulatory mechanisms for their formation and dispersal (4, 5). Genetic control of biofilms has been linked to the effects of small-molecule messengers for quorum sensing in bacterial communities (6). As a consequence, an extracellular polymeric substance is produced that determines the overall biophysical properties of a biofilm through its specific biomacromolecular composition (3). In turn, changing these macromolecular structures offers an additional control level in biofilms, for example, by matrix-degrading enzymes. For glycan-based biofilm components, regulatory glycosidases have been frequently described, in both bacterial and fungal species that can have impact on biofilm synthesis and export (7, 8, 9, 10, 11). These polysaccharide-specific enzymes can alter biofilm viscosity and thus influence the mobility of biofilm-matrix embedded or penetrating particles such as bacteria or bacteriophages, making them promising tools in antimicrobial treatments (12, 13, 14, 15, 16).

In this work, we have characterized the enzymatic modification of biofilms formed by the plant pathogen *Pantoea stewartii* subsp. *stewartii* (*P. stewartii*) (17). *P. stewartii* infects sweet corn and maize where it colonizes the xylem with dense biofilms containing the exopolysaccharide stewartan as key virulence factor. Consequently, free water flow in the plants is blocked, ultimately leading to wilting of leaves and necrosis of crops. Stewartan is an anionic heteropolymer of heptasaccharide repeat units (RU) (Fig. 1): Its backbone structure [→3)-α-d-Gal*p*^I^(1→6)-β-d-Glc*p*^II^(1→3)-β-d-Gal*p*^III^(1→] is branched at Gal^I^ with [(4→1)-β-d-GlcA*p*^IV^(4→1)-α-d-Gal*p*^V^(6→1)-β-d-Glc*p*] (18, 19). Additionally, 90% of Gal^I^ is modified by (6→1)-β-d-Glc^VI^.

**Figure 1:**
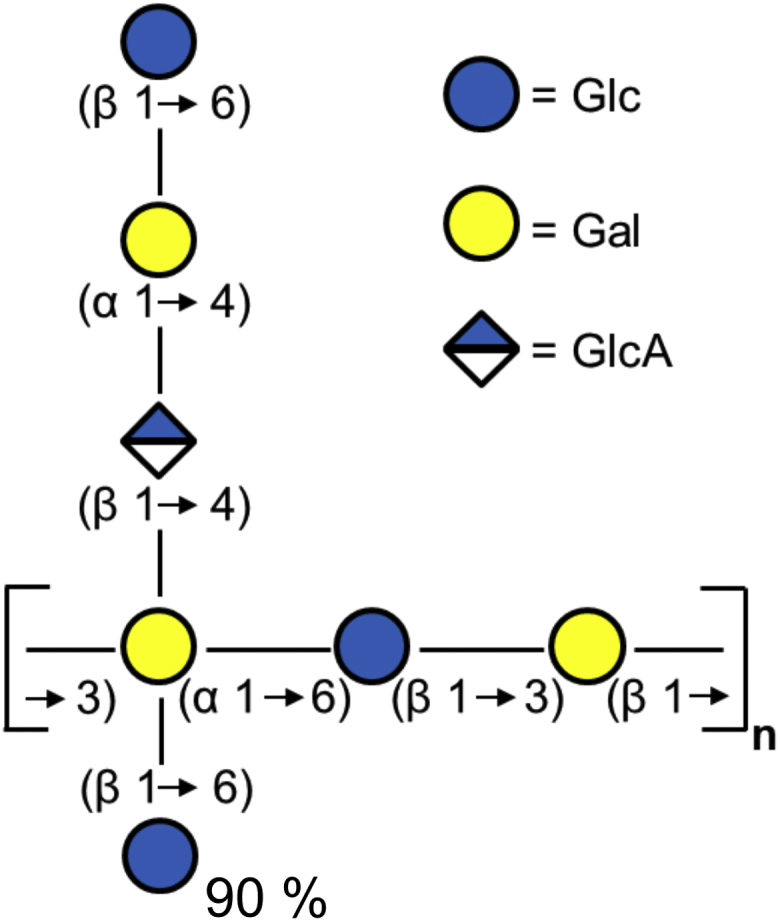
**Structure of stewartan exopolysaccharide repeat unit**. The oligosaccharide (18) is given in CFG notation (70).

Biofilm formation in *P. stewartii* is regulated by cell-density-dependent quorum sensing that controls three gene systems (19). Most stewartan biosynthetic genes are located on the *wce-I* cluster that encodes for a Wzy-dependent exopolysaccharide synthesis pathway (19, 20). Glycosyltransferases encoded by *wce-I* catalyze stewartan RU assembly. However, the glycosyltransferase gene region (*wceB, K, M, N*) contains two additional genes, *wceJ* and *wceF*. WceJ was found to be a nonfunctional pyruvate-transferase, which is not required for *P. stewartii* virulence. The *wceF* gene (formerly designated as *cpsH*) is highly conserved in *Pantoea* and *Erwinia* species (Table S1 and Fig. S1). It encodes an 80 kDa polypeptide chain (736 amino acids) following a Tat-secretion signal. A function for the corresponding protein WceF has so far not been described. Mutations in *wceF* resulted in an increased stewartan exopolysaccharide size of up to 7.6 MDa and suggested a role of WceF in stewartan chain length control (21). In this work, we report the crystal structure of WceF and its interaction with purified stewartan exopolysaccharide. WceF shows high structural similarity to bacteriophage tailspike proteins (TSPs) and is enzymatically active on stewartan, proposing a role in exopolysaccharide modification when *P. stewartii* is in the mucoid, biofilm producing state.

## Results

### WceF is a native trimer of parallel, right-handed β-helices

**Figure 2:**
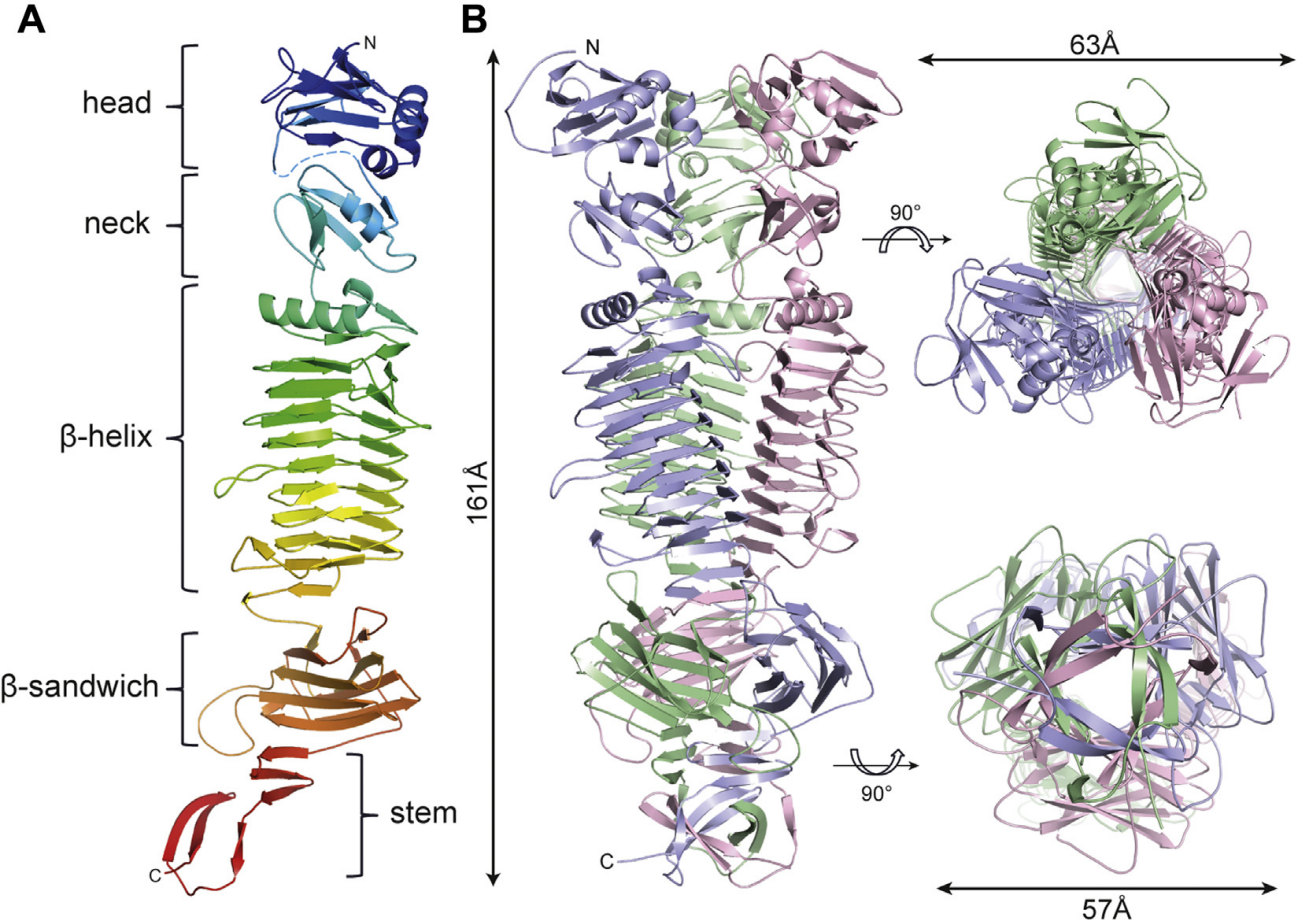
**X-ray crystal structure of *Pantoea stewartii* WceF.***A*, domain architecture of a single WceF monomer colored from N terminus (*dark blue*) to C terminus (*red*), composed of five individual domains. *B*, structure of the native trimer WceF (*side*, *top*, and *bottom view*).

We recombinantly expressed, purified, and crystallized WceF lacking the N-terminal 28 amino acids of the Tat-signal peptide that encodes for export *via* the Tat-pathway for folded proteins (22). Tat-signal peptide sequences are typically cleaved off by a signal peptidase once the protein has been transported. We therefore consider that our recombinant construct (WceF residues 29–736) represents the native form of WceF. We solved its crystal structure to 2.55 Å resolution, using a selenomethionine variant for phasing (Table S2). WceF residues 34 to 736 were resolved in the electron density. As confirmed by size-exclusion chromatography, WceF is a native homotrimer of about 200 kDa (Fig. S2). Each peptide chain is composed of an N-terminal head domain, a neck domain, the central right-handed parallel β-helix domain followed by a short triple β-helix, a β-sandwich, and a C-terminal stem domain (Fig. 2). The head domain (residues 34–146) is made of two β-sheets containing four antiparallel β-strands flanked by three α-helices. Electron density was not resolved for the following 25 residues. A five-stranded Greek-key motif surrounding one α-helix then forms the neck domain (residues 166–229) of WceF. The central body of WceF is formed by a large three-stranded β-helix (residues 236–514) of eight complete rungs capped by an N-terminal α-helix (residues 252–265). At the C-terminus of the β-helix, the chain makes a β-hairpin turn and two 120° kinks that form two turns of a triple β-helix in the native trimer (residues 517–531). The intertwined chains then again separate and individually form a β-sandwich domain with a jelly roll fold composed of eight antiparallel β-strands arranged in two four-stranded sheets (residues 531–667). At the C terminus, two smaller antiparallel β-sheets of three and two strands, respectively, are connected by a large loop (residues 678–736). All C-terminal peptide regions following the β-helix domain highly intertwine in the native trimer structure.

The WceF N-terminal head domain (residues 34–146) is classified in the Conserved Domain Database (CCD) as head domain due to its resemblance to the N-terminal domain of the TSP of bacteriophage P22 (23, 24). A structural comparison with DALI confirmed the high similarity of the WceF head domain to the P22TSP N terminus (Cα rmsd 2.6 Å) (Fig. S3) (25). The WceF neck domain, inserted between the WceF N-terminus and the β-helix domain, superimposes very well (DALI: Cα rmsd 1.1 Å) with a similar domain insert preceding the β-helix in tailspikes of bacteriophage CBA120 (26).

### WceF is structurally similar to bacteriophage tailspike proteins but lacks their high stability

An HHPred analysis identified various structural homologs of WceF (Table S3) (27). As three best hits, we identified TSPs from viunalikevirus phage CBA120 and podovirus P22 (26, 28, 29, 30). Among all hits were many bacteriophage TSP containing trimers of parallel, right-handed β-helices structurally similar to WceF (Table S3). Moreover, structural similarities with a diverse set of anionic polysaccharide degrading enzymes, mainly polygalacturonases, were confirmed for the WceF β-helix domain (residues residues 236–514).

**Figure 3:**
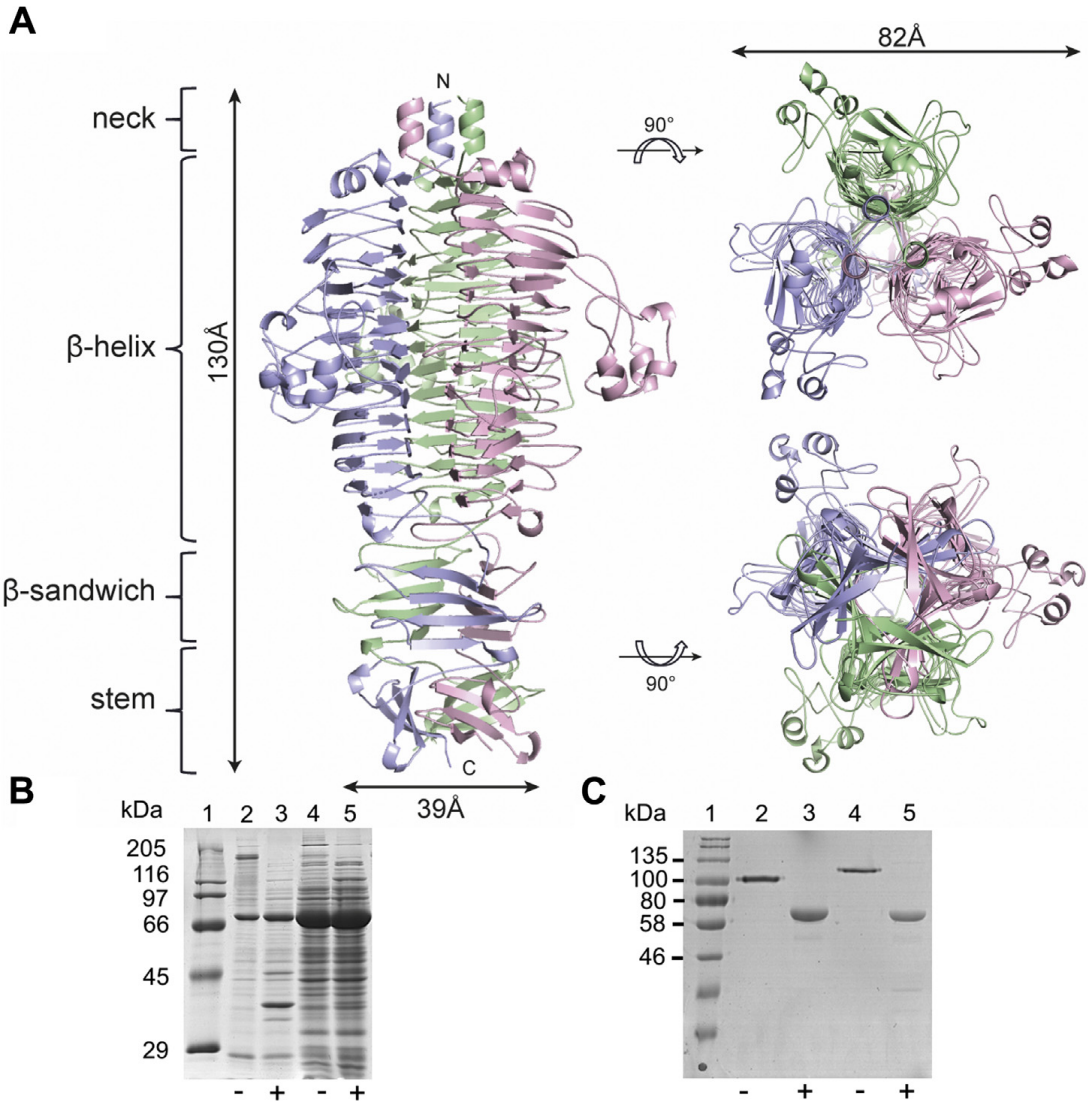
**Trimer stability of WceF compared with a bacteriophage tailspike with similar fold.***A*, structure of bacteriophage P22TSP (PDB:1TSP) (60). *B*, SDS-PAGE of WceF (80.4 kDa) after expression in *E. coli*. Lanes 2, 3: Insoluble fraction. Lanes 4, 5: Soluble fraction with (+) or without (−) heating in 2% (w/v) SDS for 5 min. *C*, purified bacteriophage HK620TSP and P22TSP do not denature in 2% SDS when not heated (31). Lanes 2, 3: HK620TSP (64.7 kDa) (51), Lanes 4,5: P22TSP (60.1 kDa) with (+) or without (−) heating in 2% (w/v) SDS for 5 min.

Bacteriophage TSPs are thermostable enzymes to ensure bacteriophage infectivity even under harsh extracellular conditions (31). Their native trimeric β-helix assemblies are kinetically stabilized, resulting in slow denaturation at temperatures above 70 °C, even in the presence of detergents. In contrast, and in spite of its trimeric assembly of β-helices similar to the one found in TSPs, WceF rapidly denatured in the presence of 2% (w/v) SDS even at room temperature (Fig. 3*B*). WceF migrated as a monomer in SDS-PAGE, whereas the TSPs of bacteriophages P22 and HK620 remained native trimers in the presence of SDS. As shown before, P22TSP only denatured into monomers after heating to 100 °C for several minutes prior to electrophoresis (32). Hence, the bacterial protein WceF is notably less stable in the presence of detergent compared with the P22TSP of bacteriophage origin. This is in agreement with a calculated overall lower WceF trimer interface stabilization (Fig. S9). For P22TSP and WceF, similar trimer interface areas were found; however, due to their markedly different surface properties, the WceF trimer interface was predicted to contribute less stabilization compared with the P22TSP interface.

We produced N-terminally truncated WceF variants to assess their influence on trimer stabilization (Fig. S4). Constructs lacking only the P22TSP-homologous head domain (residues 34–146) were soluble but rapidly aggregated, presumably because they were impaired in trimer stabilization. Constructs lacking more N-terminal residues, *i.e.*, the neck domain or the α-helix cap preceding the β-helix were insoluble. This is in contrast to P22TSP, where the N-terminal head domain was dispensable for trimer stabilization (33), we hence conclude that Wcef is a stable native trimer only in the presence of all its N-terminal domains.

### WceF cleaves stewartan exopolysaccharide

Depolymerization activity on polysaccharide capsules, exopolysaccharide biofilms, or lipopolysaccharide is typically found in bacteriophage TSPs where it can essentially drive the infection process (34, 35). Moreover, right-handed, parallel β-helices of prokaryotic origin have been often found to be involved in glycan binding (36). We therefore tested WceF interactions with stewartan as the *P. stewartii* major biofilm exopolysaccharide component. Stewartan isolated from three-day-old *P. stewartii* biofilms is highly viscous in solution (14).

**Figure 4:**
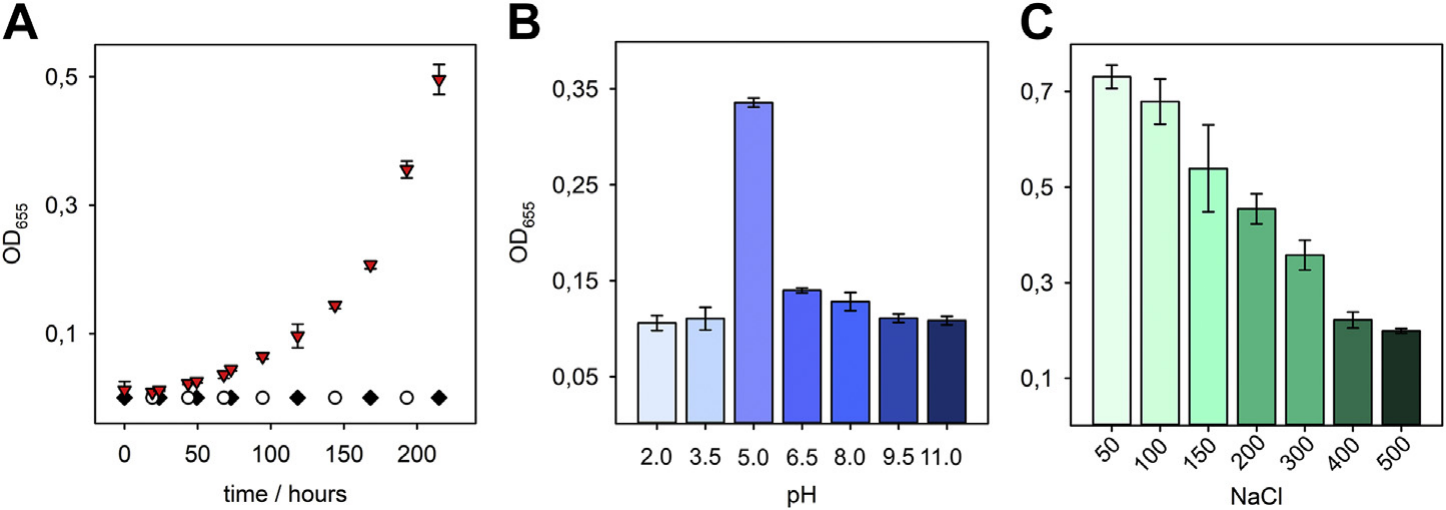
**WceF cleaves stewartan exopolysaccharide.** Enzymatic activity of WceF (1.5 μM) at 30 °C on the stewartan exopolysaccharide (1 mg ml^−1^) analyzed by reducing end formation monitored with 3-methyl-2-benzothiazolinon-hydrazone (MBTH) (37). *A*, time course of reducing end increase in WceF–stewartan mixtures (*red triangles*) or stewartan (*white circles*) or WceF alone (*black diamonds*) in 50 mM MES, pH 5, 50 mM NaCl. *B* and *C*, reducing end formation from stewartan after 120 h incubation with WceF at varying pH values (*B*) or NaCl concentrations (*C*). All error bars show standard deviations obtained from three independent measurements.

However, when mixed with WceF, stewartan solutions lost their viscosity after about 3 days at room temperature (Fig. S5). From this, we assumed stewartan cleavage and analyzed WceF-stewartan mixtures for reducing end formation (Fig. 4*A*). Reducing ends were produced slowly over a time course of more than eight days only, tested with the 3-methyl-2-benzothiazolinon-hydrazone (MBTH) method (37). However, after this time, we observed a sudden, about 30-fold increase in reducing end formation velocity. WceF or stewartan alone did not produce reducing ends over the whole 300 h time course of the experiment. WceF showed highest activity at pH 5 (Fig. 4*B*) that decreased with increasing salt concentrations (Fig. 4*C*). No absorption increase at 235 nm was found during the whole experiment time as it would be characteristic for double bond formation due to a putative polysaccharide lyase activity. We thus conclude that WceF is a glycoside hydrolase specific for stewartan.

### A stewartan-specific bacteriophage tailspike produces oligosaccharides more rapidly than WceF

**Figure 5:**
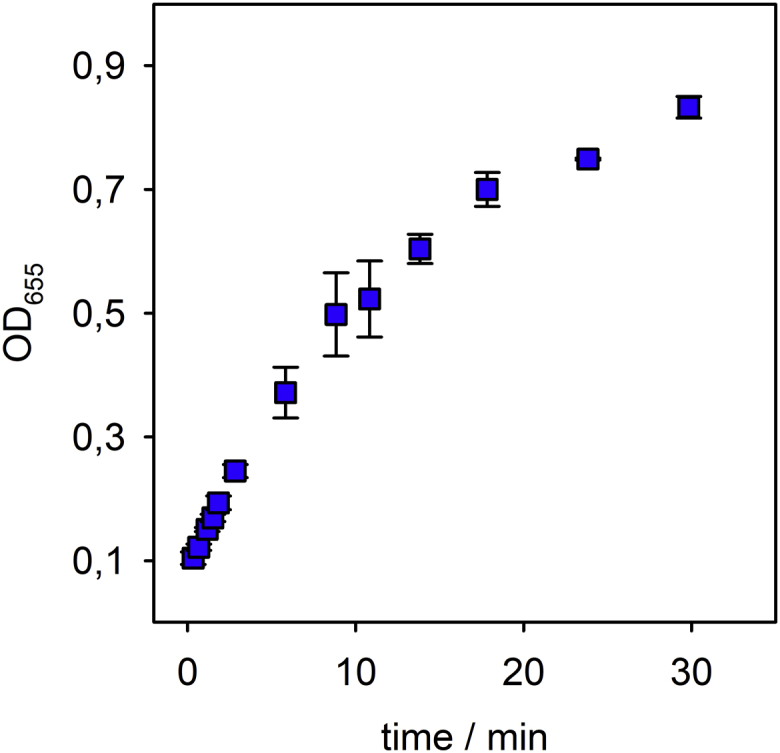
**A stewartan-specific bacteriophage tailspike produces oligosaccharides more rapidly than WceF**. Activity of bacteriophage φEa1H tailspike protein (0.14 μM) on stewartan exopolysaccharide (1 mg ml^−1^) analyzed by following reducing end formation with the MBTH test. Standard deviations from three independent experiments.

*P. stewartii* is host for a number of bacteriophages. To compare WceF with a bacteriophage enzyme, we analyzed the tailspike depolymerase from podovirus φEa1H. φEa1HTSP shares about 25% sequence identity with WceF (38). φEa1HTSP cleaved stewartan at significantly higher velocity than WceF, *i.e.*, the enzymatic formation of reducing ends from stewartan reached saturation within 30 min (Fig. 5). Moreover, φEa1HTSP was active over a broad pH range between 5 and 10 and independent of the salt concentration (Fig. S6). Also, we had to use a tenfold lower φEa1HTSP enzyme concentration (0.14 μM) compared with the experiments with WceF (1.50 μM) to be able to follow the time course of the enzymatic reaction. The bacteriophage depolymerase φEa1HTSP was therefore notably more efficient than the bacterial enzyme WceF. As described earlier, we used φEa1HTSP to produce stewartan oligosaccharides of one and two repeat units, respectively (Fig. S7) (39). φEa1HTSP cleaves the φ-1,3-glycosidic bond of two backbone galactoses in stewartan (*cf.* Fig. 1).

**Figure 6:**
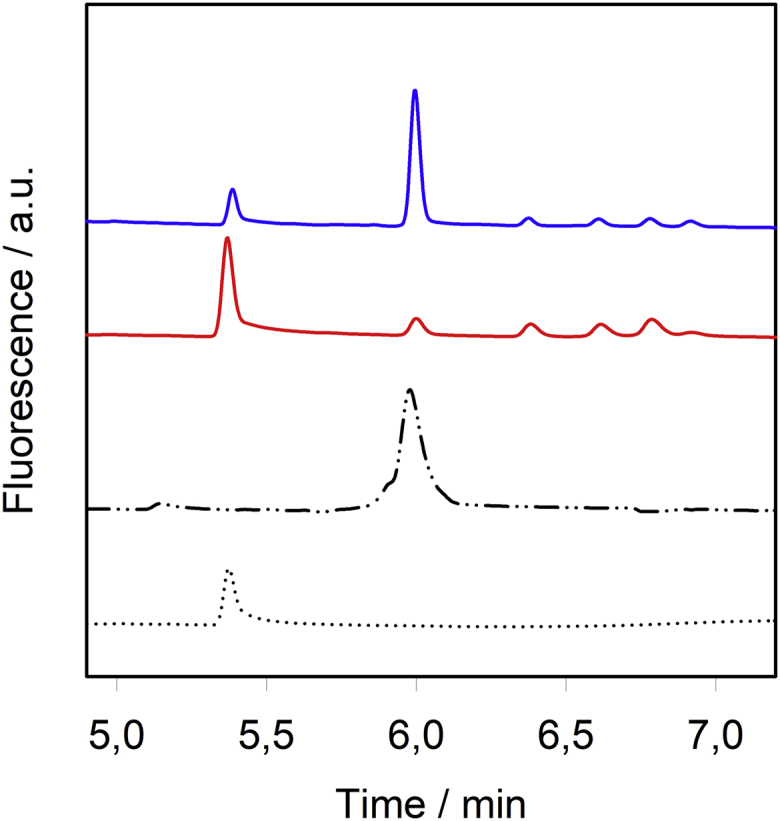
**Cleavage products after incubation of stewartan with WceF or ΦEa1hTSP.** Capillary electrophoresis analysis of stewartan exopolysaccharide (1 mg ml^−1^) cleavage products after treatment with WceF (197 h, *red*) or ΦEa1hTSP (6 min, *blue*). Oligosaccharides were purified from stewartan hydrolysis with ΦEa1hTSP (*cf.*Fig. S7) and used as 1RU (*dotted*) or 2RU (*dash-dotted*) standards for peak assignment.

We used these oligosaccharides as standards in capillary electrophoresis (CE) to analyze stewartan cleavage products obtained by either WceF or φEa1HTSP (Fig. 6). Due to the different glycosidic bond cleavage velocities found for both enzymes, we compared oligosaccharide products after different reaction times, *i.e.*, 197 h for WceF and 6 min for φEa1HTSP, respectively. From the very similar CE elution profiles compared with the stewartan 1RU standard, we conclude that WceF had depolymerized stewartan to 1RU heptasaccharides (7mers). In contrast, after the short reaction time, φEa1HTSP had mainly produced 2RU tetrakaidekasaccharides (14mers). Analytical size-exclusion chromatography and MALDI-MS analysis further confirmed that WceF had produced 1RU fragments of stewartan (Fig. S7). We therefore propose that WceF, like φEa1HTSP, is a β-1,3 galactosidase.

### WceF forms aggregates in the presence of stewartan

**Figure 7:**
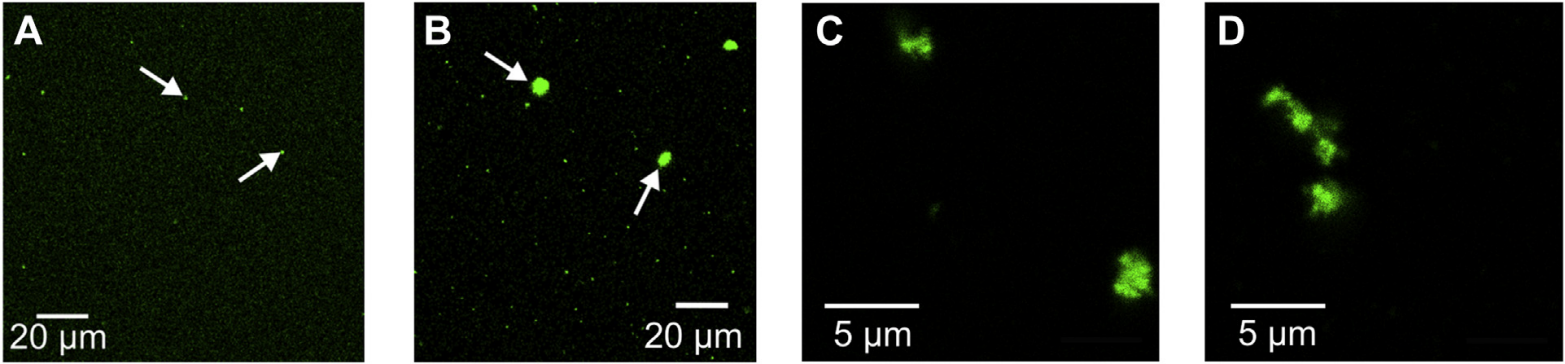
**WceF forms aggregates in the presence of stewartan.** Fluorescence microscopy image of ATTO 488-labeled WceF in (*A*) buffer (50 mM MES, pH 5, 50 mM NaCl) or (*B* and *C*) 1 mg ml^−1^ or (*D*) 20 mg ml^−1^ stewartan.

Concentrated stewartan solutions at about 10 mg ml^−1^ had shown earlier a notable diffusion hindrance for nanoparticles (14). Native trimers of WceF are large proteins (ca. 200 kDa, *cf.* Fig. S2) that may diffuse only slowly in viscous stewartan solutions. As WceF indeed exhibited a very slow initial phase of reducing end formation (*cf.* Fig. 4), we analyzed the diffusion behavior of an ATTO 488-labeled WceF with fluorescence microscopy (Fig. 7). We found that in the absence of stewartan, WceF was slightly prone to aggregation, as indicated by the presence of small fluorescent spots. However, when WceF was mixed with stewartan, we observed much larger fluorescent spots with an estimated diameter of 3 to 10 μm, indicating that WceF had associated into roughly spherical protein aggregates. Particles of this size are too large to freely diffuse in stewartan (14). Stewartan solutions at about 1 mg ml^−1^ hence were able to autoagglutinate WceF. WceF, while binding to stewartan, however, does not mask cleavage sites for the stewartan depolymerase φEa1HTSP. Free diffusion of phage particles in the stewartan matrix was restored equally well both in the presence and in the absence of WceF, as shown by fluorescence correlation spectroscopy (14) (Fig. S8).

## Discussion

Glycan-modifying proteins have been regularly described in the synthesis and export machines that control bacterial and fungal extracellular polysaccharides involved in encapsulation or biofilm formation. In this respect, the presence of enzymes that cleave glycosidic bonds seems counterintuitive, and, indeed, biochemical characterization of these proteins combined with mutagenesis studies points to their multifaceted functional roles in polysaccharide modification (7, 8, 9, 10, 40). This work showed that *Pantoea stewartii* WceF, as part of the biofilm synthesis operon, is a hydrolytic enzyme, active on *P. stewartii’s* biofilm exopolysaccharide stewartan, and with a bacteriophage tailspike-like fold.

### WceF has a fold reminiscent of a bacteriophage tailspike protein but is less stable

WceF is a native trimer of parallel, right-handed β-helices. This fold is widespread in proteins from predominantly prokaryotic species that are involved in binding and enzymatic modification of polysaccharides (36). Parallel, right-handed β-helices occur in monomeric proteins, such as in pectate lyases (41), or as oligomers, such as in trimeric bacteriophage TSPs (42). A monomeric β-helix fold has been found in VexL of *Achromobacter denitrificans*, a hydrolase involved in capsular polysaccharide modification (10), but in general, biofilm or capsule-modifying enzymes of fungal or bacterial origin can be structurally diverse (7, 43, 44).

To our knowledge, *P. stewartii* WceF is the first enzyme of this type that occurs as a β-helix trimer. So far, these trimers have only been found as part of bacteriophage TSPs. TSPs are elongated fibrous proteins that serve in glycan receptor recognition and cleavage to start the infection cycle in tailed bacteriophages (35, 45, 46). TSP structures contain defined building blocks, with N-terminally conserved domains for specific assembly with other proteins of the tail machinery and C-terminal parts that serve in host glycan receptor recognition (26, 47). Remarkably, not only the trimeric WceF β-helix, but also its N-terminal domain and the neck domain inserted before the β-helix are structurally homologous to similar domains in bacteriophage TSPs. It is thus tempting to speculate that *P. stewartii* has acquired WceF during coevolution with bacteriophages to serve in modification of biofilm glycans, although the folds may have functionally diversified. The structural homolog of the WceF N-terminal domain found in bacteriophage P22TSP serves in capsid assembly, but is dispensable for protein assembly or trimer stabilization (33). Hence, P22TSP, lacking this domain, is stable and highly soluble.

However, WceF variants lacking this N-terminal domain were aggregation prone, and we were unable to assess the role of this domain for WceF trimer stabilization. This means that WceF is not kinetically stabilized, and it readily unfolds in the presence of detergents, in contrast to the high stability reported for many TSPs (31). The specific WceF single chain fold organization and trimer interfaces may account for these stability differences. For example, in the WceF β-helix, the first N-terminal rung lacks a typical short turn, but has a long loop insertion that disturbs a parallel alignment of the β-helices, resulting in a less tightly packed trimer interface compared with P22TSP. Also at the intertwined C terminus, different intermolecular interactions might alter trimer stabilization. Here, WceF has salt-bridge-interconnected sandwich domains, whereas P22TSP forms a hydrophobic core between β-prism domains (Fig. S9).

### WceF is a hydrolytic enzyme of stewartan exopolysaccharide

*In vitro*, our analyses showed that WceF degraded stewartan rather slowly. In contrast, stewartan enzymatic hydrolysis was much faster with the ΦEa1hTSP depolymerase of bacteriophage origin. WceF featured a pronounced lag phase, and reducing end production only accelerated after several days, probably because WceF formed aggregates in the presence of stewartan. From the WceF hydrodynamic radius, we estimate that the size of these highly dense protein clusters corresponds to roughly 1000 WceF molecules that can form in a 1 mg ml^−1^ stewartan solution. Previous work showed that stewartan may act as a molecular sieve in which diffusion is limited by both concentration-dependent polysaccharide chain entanglement and noncovalent interactions with the matrix (14). We hence assume that WceF forms clusters by association with stewartan. Exopolysaccharides in general provide multivalent interfaces for electrostatic and hydrophobic interactions (48). The WceF trimer could thus cross-link stewartan in analogy to lectins (49), although we did not observe a decrease of stewartan solubility or even precipitation in the presence of WceF.

Furthermore, the long-lasting attachment of WceF with its substrate might be indicative for a processive mode of stewartan hydrolysis (50). Processivity usually is a trade-off between a high affinity of the enzyme toward the substrate and a low hydrolysis velocity, which is in agreement with the low hydrolytic activity seen for WceF. WceF might remain bound to the stewartan polymer and move along the chain while enzymatically releasing single stewartan RUs. As a result, the long chains of stewartan would be degraded in a stepwise fashion. In agreement with WceF acting as an endo-enzyme, size-exclusion chromatography of reaction products showed longer oligosaccharides. In contrast, ΦEa1hTSP stewartan hydrolysis is marked by less processivity and a rapid accumulation of short oligosaccharides. Previous fluorescence correlation spectroscopy analyses showed that ΦEa1hTSP restored diffusion of large particles in the stewartan matrix in less than 30 min, a prerequisite for the phage to penetrate the biofilm (14). Like bacteriophage TSPs, also WceF produces oligosaccharides that correspond to at least single RU of its glycan heteropolymer substrate. Its binding site thus must accommodate at least one RU (51). From this it follows that WceF, like other exopolysaccharide depolymerases, most probably is inactive in the cytosol, where RUs are synthesized but not assembled to polymers, further stressing a role of WceF for extracellular glycan modification.

### What is the function of WceF in *Pantoea stewartii* biofilms?

The conservation of WceF homologs in many *Pantoea* species points to a functional role of these proteins that is so far not fully understood. WceF contains a twin-arginine translocation signal for inner membrane translocation; however, in this work we have not analyzed its final localization. Extracellularly, WceF aggregate formation with stewartan, as observed in this work, could strengthen the biofilm matrix by protein–glycan cross-links. Clustering of colloidal particles with other polymer components is a common feature found in other bacterial biofilms, *i.e.*, cell clumping in the extracellular matrix of *S. epidermidis* or clustering of fluorescent beads with curli fiber components in *E. coli* biofilms (16, 52, 53). In these cases, clusters of cells or beads had a size (1–5 μm) similar to the WceF clusters seen in this work.

In general, chain length and concentration determine a polymer’s physical properties in solution, for example, critical entanglement concentrations and thus its properties as a diffusion-limiting molecular sieve matrix (54). Hence, trimming chain lengths of secreted exopolysaccharides by endoglycanases can be important to control external glycan properties (8, 10, 55). Presence of *wzc* in the WceF stewartan biosynthesis cluster suggests the typical, Wzz-like chain length regulation. Wzz was proposed to inhibit the Wzy polymerase by transmembrane domain interactions, controlled by polysaccharide interactions with its oligomeric, periplasmic coiled-coil domains (56). *P. stewartii* *wceF* knockouts were found to increase the stewartan chain lengths (21). We may thus speculate that WceF is hydrolytically active on stewartan either during periplasmic transfer, eventually cleaving off parts of growing chain. Alternatively, WceF activity in the biofilm may regulate the mean chain length distribution toward shorter chains to modify its overall microviscosity (14). Current studies in our laboratory aim at setting up antibody-based detection systems for WceF to monitor its presence under different *P. stewartii* growth conditions, also inside attacked plants.

## Conclusion

Structure and biochemistry of the protein WceF, encoded by the exopolysaccharide synthesis cluster of *P. stewartii*, emphasize that specific protein interactions may regulate biofilm glycans. Biofilm functionality depends on its character as a viscous matrix that can act as a mechanical scaffold and a molecular sieve. Polymer chemistry of the biofilm’s components determines these biophysical properties (3). WceF, but also phage hydrolases, that are active on polymeric glycan substrates thus act in biofim matrix modification. Addressing glycan polymers in biofilms by bacterial glycosidases has impact on biofilm dispersal also in other systems (5, 11). Here, *P. stewartii* WceF serves as a model structure for a series of protein homologs present in other plant pathogens of economic importance that attack pineapples, apples, onions, or rice (Table S1). Future studies will focus on functionally understanding these interactions, also to pave the way for modulation of biofilm properties with engineered glycanases for crop pathogen control or therapeutic applications (1, 57, 58, 59).

## Experimental procedures

### Materials

*Pantoea stewartii* subsp. *stewartii* DSM 30176 was purchased from the German Collection of Microorganisms and Cell Cultures (DSMZ). Stewartan preparation and purification have been described elsewhere (14). A *c*DNA containing the gene for bacteriophage φEa1h TSP depolymerase was obtained from K. Geider (Julius KühnInstitut, Dossenheim, Germany) (38). Cloning and purification of P22TSP and HK620TSP have been described earlier (51, 60). If not indicated otherwise, 50 mM HEPES-NaOH, 200 mMNaCl, pH 8.5 was used as the standard buffer. All chemicals were of analytical grade, and ultrapure water was used throughout.

### Cloning and protein purification

WceF was amplified from *P. stewartii* genomic DNA using oligonucleotide forward primer 5'-CGATATCCCAACGACCGAAAACCTGTATTTTCAGGGCGCCAAAAATACAGGTCTGG and reverse primer 5'-GCCGCGAAGCTTACGCTTTAATGGC. The same reverse primer and the forward primer 5'-CGCGCATATGCATCACCATCACCATCACGATTACGATACCCAACGACCGAAAACC were then used to introduce a His_6_ tag followed by a TEV-protease cleavage site. The resulting fragment was cloned into a pET23a plasmid, using NdeI and HindIII restriction sites. WceF lacks the N-terminal Tat signal peptide (aa 28–736). After protein expression in *E. coli* BL21(DE3) at 20 °C and French press cell rupture, WceF in the soluble fraction was further purified with immobilized metal affinity chromatography (IMAC). The His_6_ tag was cleaved off with His_6_-TEV protease and tag-free proteins collected in the flow-through of a second IMAC chromatography. Similarly, a selenomethionine (SeMet) variant was prepared with the protein expressed in *E. coli* B834(DE3) as described (61). The final protein preparations exhibited homogeneities of more than 95% as estimated from SDS-PAGE with silver staining and with a yield of typically 5.1 to 8.9 mg protein per g of wet cells. For fluorescence microscopy, WceF was labeled with ATTO 488 NHS-Ester according to manufacturer protocols (ATTO-Tec, Siegen, Germany). Average labeling yield was 1.59 dye molecules per protein, calculated from the 550/280 nm absorption ratio.

### Crystallization, diffraction data collection, and structure determination

WceF was crystallized using the sitting-drop vapour-diffusion method at 20 °C using a Gryphon pipetting robot (Art Robbins Instruments) and Rock Imager storage system (Formulatrix). In total, 200 nl SeMet-substituted WceF (10 mg ml^−1^) or native WceF (8 mg ml^−1^) was mixed with an equal volume of reservoir solution containing 18% PEG2000 MME, 100 mM ammonium sulfate, and 100 mM sodium acetate buffer, pH 4.6 (SeMet) or 20% PEG3350, 200 mM di-ammonium-hydrogen citrate, pH 5.0 (native). Crystals appeared within 1 week, were transferred in reservoir solution containing 20% ethylene glycol, and flash-cooled in liquid nitrogen. Data sets were recorded at 100 K from single crystals at BESSY II, Berlin, BL14.1 (0.9798 Å, SeMet), or BL14.2 (0.9184 Å, native) and processed and scaled using the XDS program suite (62, 63). The asymmetric unit contained three protein chains and 54% solvent, Se sites (18 of 24) were detected by Autosol/PHENIX (64). The density showed a mostly continuous trace for the peptide backbone and clear anomalous signals for SeMet positions. An initial model was manually built using COOT and iteratively refined with Refmac, including noncrystallographic symmetries (65, 66). The improved model was used for molecular replacement with Phaser and refined with Refmac against the native 2.55 Å data to a final R_work_ and R_free_ of 22.42% and 26.93%, respectively (67). The model was confirmed with MolProbity with 94% of the residues lying in allowed Ramachandran plot regions (68). The final coordinates were deposited in the Protein Data Bank with accession number 6TGF. Figures were generated with PyMOL (version 2.0, Schrödinger, LLC).

### Enzyme activity analyses

Stewartan was digested by adding 0.14 μM ΦEa1h TSP or 1.5 μM WceF to 1 mg ml^−1^ stewartan in 50 mM MES-NaOH, 100 mM NaCl, pH 5. Increase of reducing end concentration was monitored with MBTH as described (37). For capillary electrophoresis, the reaction was stopped at indicated time points by adding ethanol (80% v/v). Samples were centrifuged, and the supernatant was dried and dissolved in 1.5 μl 200 mM 8-aminopyrene-1,3,6-trisulfonic acid in 15% (v/v) acetic acid. In total, 1.5 μl sodium cyanoborhydride in 1 M tetrahydrofuran was added and incubated overnight at 37 °C. Samples were diluted to 100 μl with water and subjected to capillary electrophoresis as described elsewhere (69).

### Confocal fluorescence microscopy

Fluorescence microscopy images were acquired on a Zeiss LSM780 system (Carl Zeiss, Oberkochen, Germany) using a 40×, 1.2 NA water immersion objective. Samples were excited with a 488 nm argon laser. Excitation and detection light were separated by a 488 nm dichroic mirror and fluorescence was detected between 500 and 600 nm, using a GaAsP detector in photon counting mode.

## Data availability

All of the data are contained within the main paper and Supporting Information.

## Conflict of interest

The authors declare that they have no conflicts of interest with the contents of this article.
