# Supplementary material for: Pantoea stewartii WceF is a glycan biofilm-modifying enzyme with a bacteriophage tailspike-like fold
Source: J Biol Chem. 2021 Jan 13;296:100286. doi: 10.1016/j.jbc.2021.100286 (PMC7949094; doi:10.1016/j.jbc.2021.100286)
Supplement: Supplemental Figures and Tables [file mmc1.pdf]

**SUPPORTING INFORMATION**

# *Pantoea stewartii* WceF is a glycan biofilm modifying enzyme with a bacteriophage tailspike-like fold


Tobias Irmscher[1,2,#], Yvette Roske[3,6,#], Igor Gayk[1], Valentin Dunsing[4], Salvatore Chiantia[4], Udo Heinemann[3,5,*], and Stefanie Barbirz[1,*]

[1]Physikalische Biochemie, Universität Potsdam, Karl-Liebknecht-Str. 24-25, 14476 Potsdam, Germany.

[2]Max Planck Institute of Colloids and Interfaces, Am Mühlenberg 1, 14476 Potsdam, Germany.

[3]Max-Delbrück-Centrum für Molekulare Medizin, Robert-Rössle-Str. 10, 13125 Berlin, Germany.

[4]Physikalische Zellbiochemie, Universität Potsdam, Karl-Liebknecht-Str. 24-25, 14476 Potsdam, Germany.

[5]Institut für Chemie und Biochemie, Freie Universität, Takustr. 6, 14195 Berlin, Germany.

#Authors contributed equally

*Corresponding authors: Stefanie Barbirz, Udo Heinemann

Email: barbirz@uni-potsdam.de, heinemann@mdc-berlin.de


**Running title:** Phage tailspike-like biofilm glycosidase from P. *stewartii*

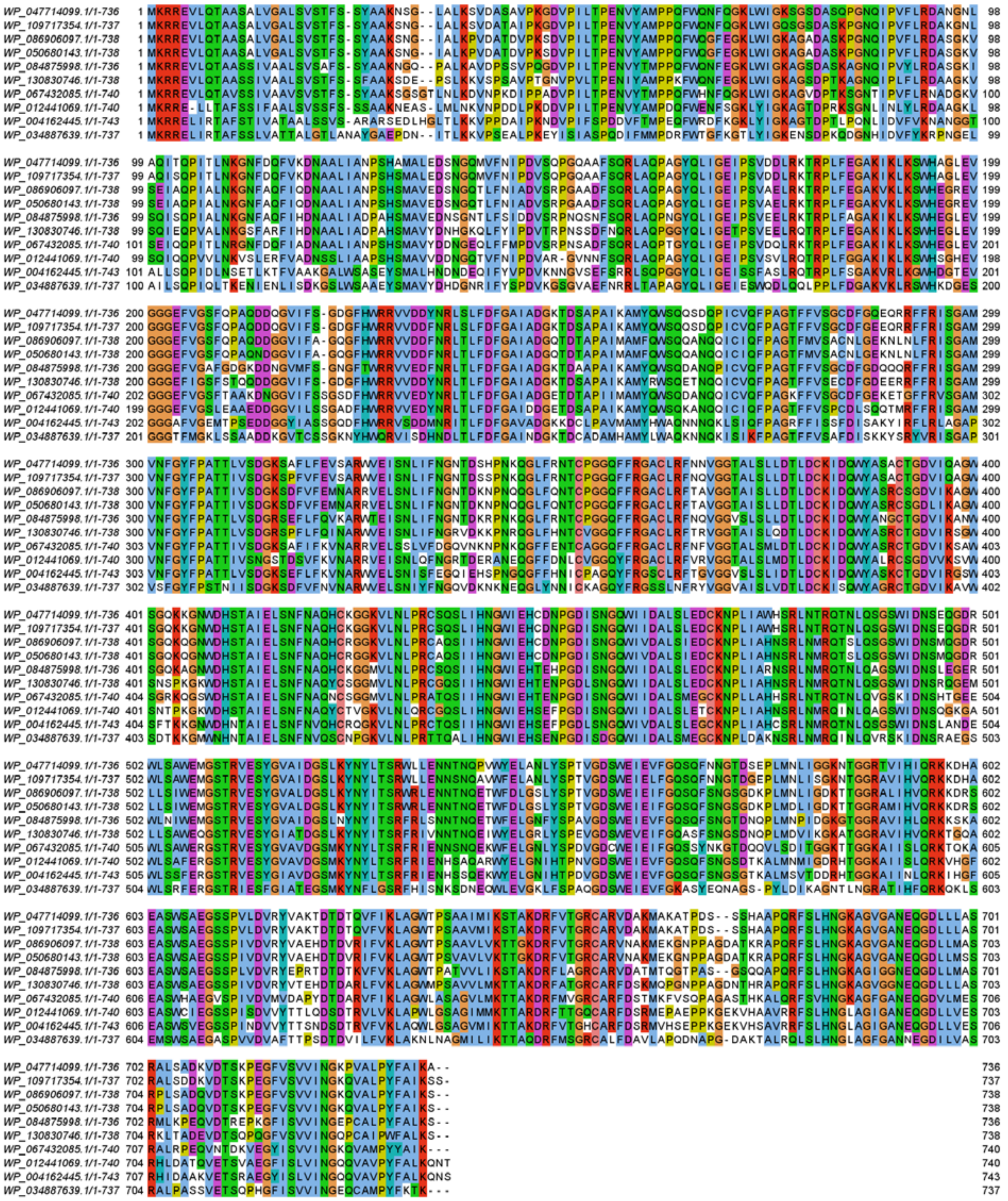

**Figure S1: Sequence alignment of conserved, WceF-like proteins within *Erwinia* and *Pantoea* species (see also Table S1).** Sequences were aligned with Clustal with default settings ("Clustal W and Clustal X version 2.0", Bioinformatics, 23: 2947 (2007)).

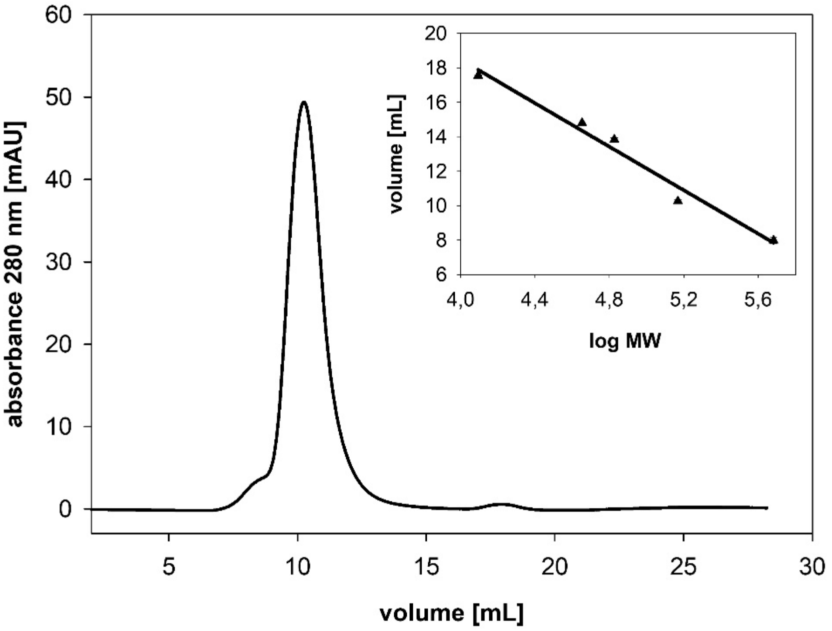


**Figure S2: Size exclusion chromatography of native WceF.** WceF was analyzed on a Superdex® 200 10/300. The inset shows a calibration curve using ferritin (equine, 480 kDa), aldolase (rabbit, 147 kDa), albumin (bovine, 67 kD), albumin (chicken, 45 kDa) and cytochrome C (equine, 12.4 kDa). WceF eluted at 10.2 mL, resulting in an apparent molecular weight of 204.2 kDa for the native trimer (a single WceF chain has a calculated MW of 77.7 kDa).

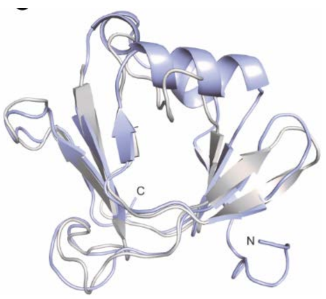


**Figure S3: Comparison of WceF head domain with bacteriophage P22 head domain.** Superposition of WceF head domain (aa 47-149) in light blue onto the P22 tailspike (PDB: 2XC1) head domain (aa 26-104) in gray, showing a similar overall fold (rmsd 1.27 Å).

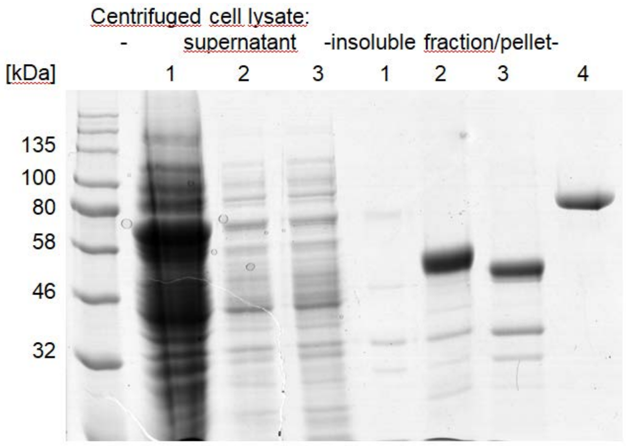


**Figure S4: SDS PAGE of soluble and non-soluble fractions after cell lysis of E. coli BL21(DE3) expression of N-terminally truncated WceF constructs.** (1) WceF lacking the head domain (residues 147-736, 67.3 kDa), (2) the neck domain (residues 230-736, 57.8 kDa) or (3) the β-helix capping domain (residues 266-736, 54.0 kDa). (4) A purified sample of purified, soluble full-length WceF (residues 29-736, 80.3 kDa) is shown for comparison.

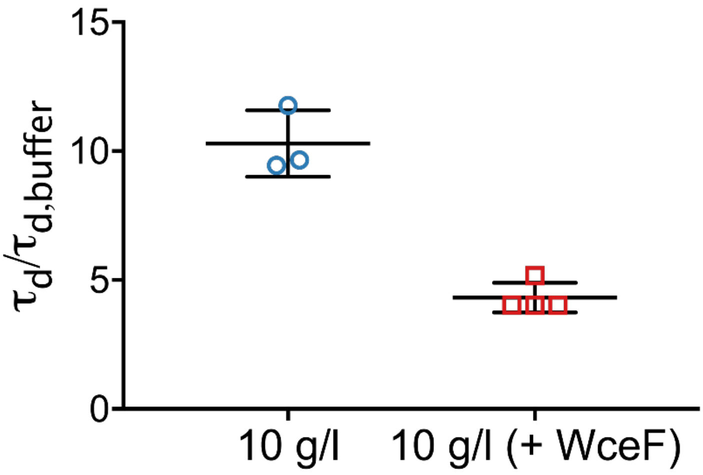


**Figure S5: WceF reduces the viscosity of stewartan solutions after 75 h at room temperature.** Fluorescence correlation spectroscopy was used to analyze hindrance of 60 nm latex beads (τd/τd,buffer) in the stewartan matrix (10 g/l) in absence (blue) or presence of WceF (red) (for a description of the method see Dunsing, V.; Irmscher, T.; Barbirz, S.; Chiantia, S. Purely Polysaccharide-Based Biofilm Matrix Provides Size-Selective Diffusion Barriers for Nanoparticles and Bacteriophages. *Biomacromolecules* **2019**, *20*, 3842-3854, doi:10.1021/acs.biomac.9b00938.).

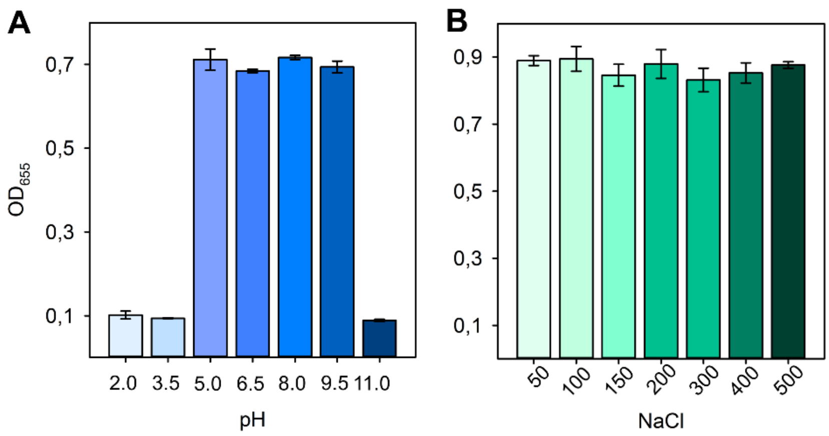


**Figure S6: Activity of ϕEa1h TSP at varying pH and salt concentrations**. ΦEa1h TSP (141 nM) was incubated with 1 mg ml$^{-1}$ stewartan for 72 h at varying pH (A) or salt concentration (B).

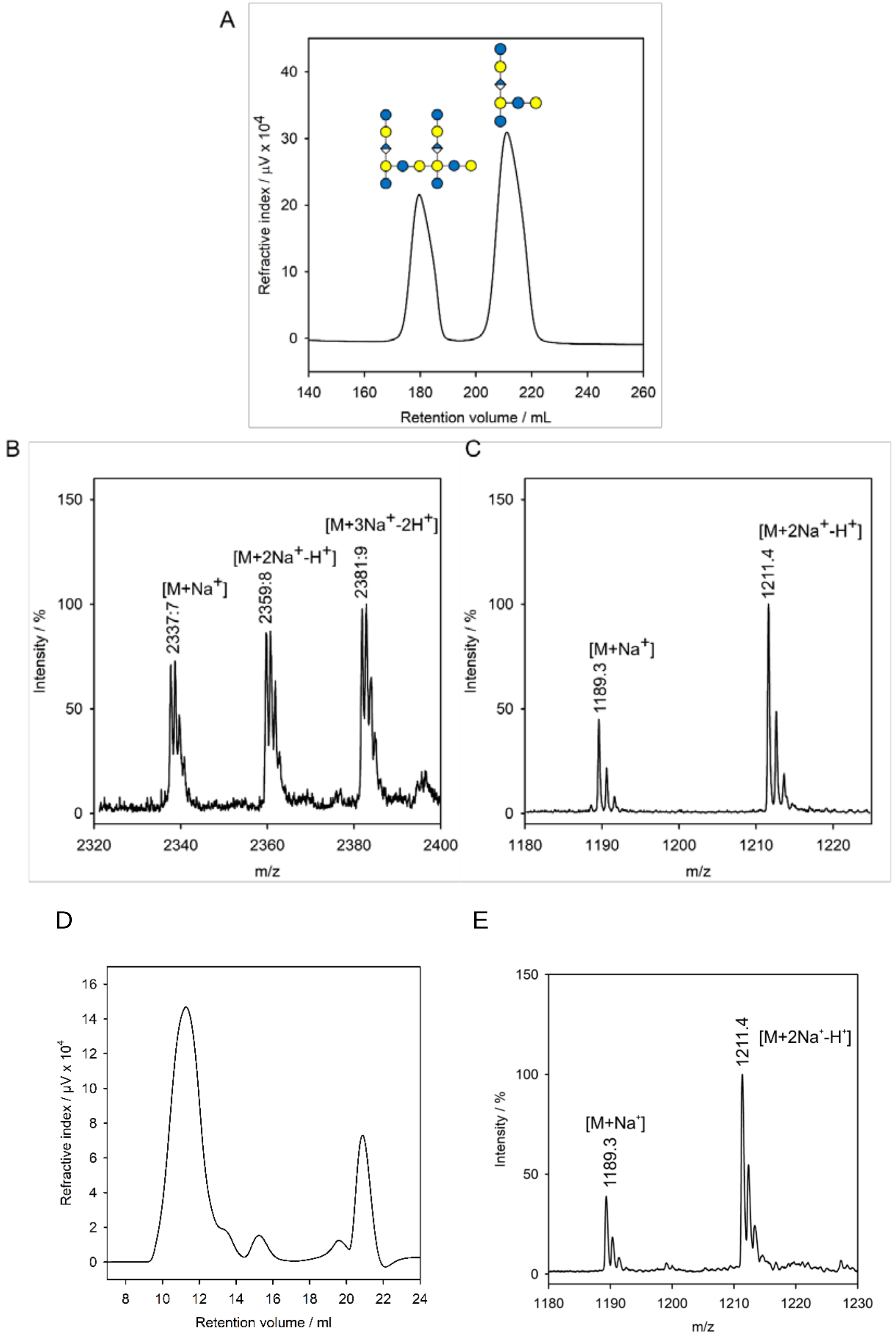


**Figure S7: Size exclusion chromatography and MALDI-MS analysis of stewartan exopolysaccharide digests with ΦEa1h TSP or WceF.** (A) Size-exclusion chromatography (SEC, Superdex® 30 26/60) after stewartan cleavage by ΦEa1h TSP. (B-C) MALDI-MS spectra of oligosaccharide fragments. Fractions pooled after SEC at ca. 180 ml (B) or ca. 215 ml (C) revealed masses corresponding to two RU (B) or one RU (C), respectively. (D) SEC (Superdex® 30 10/300) of stewartan after 197 h of incubation with WceF.  (E) MALDI-MS of WceF-stewartan digest fraction pooled 15 ml corresponding to one RU. Eluent in all SEC runs was 100 mM acetic acid.

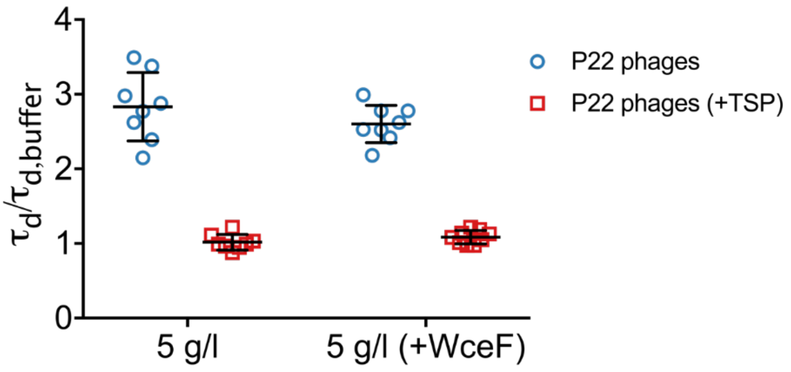


**Figure S8: WceF does not hinder access of bacteriophage ΦEa1h TSP depolymerase to cleavage sites in stewartan.** Fluorescence correlation spectroscopy was used to analyze hindrance of particles (τd/τd,buffer) in the stewartan matrix (5 g/l) (for a description of the method see Dunsing, V.; Irmscher, T.; Barbirz, S.; Chiantia, S. Purely Polysaccharide-Based Biofilm Matrix Provides Size-Selective Diffusion Barriers for Nanoparticles and Bacteriophages. *Biomacromolecules* **2019**, *20*, 3842-3854, doi:10.1021/acs.biomac.9b00938.) Bacteriophage particles (0.2 nM are hindered by the matrix (blue circles), but addition of the TSP (φEa1h depolymerase, 141 nM) cleaves stewartan and restores free diffusion of phage particles (red squares). When we did this experiment in the presence of WceF (1.5 µM), the TSP still could restore free diffusion of the phages.

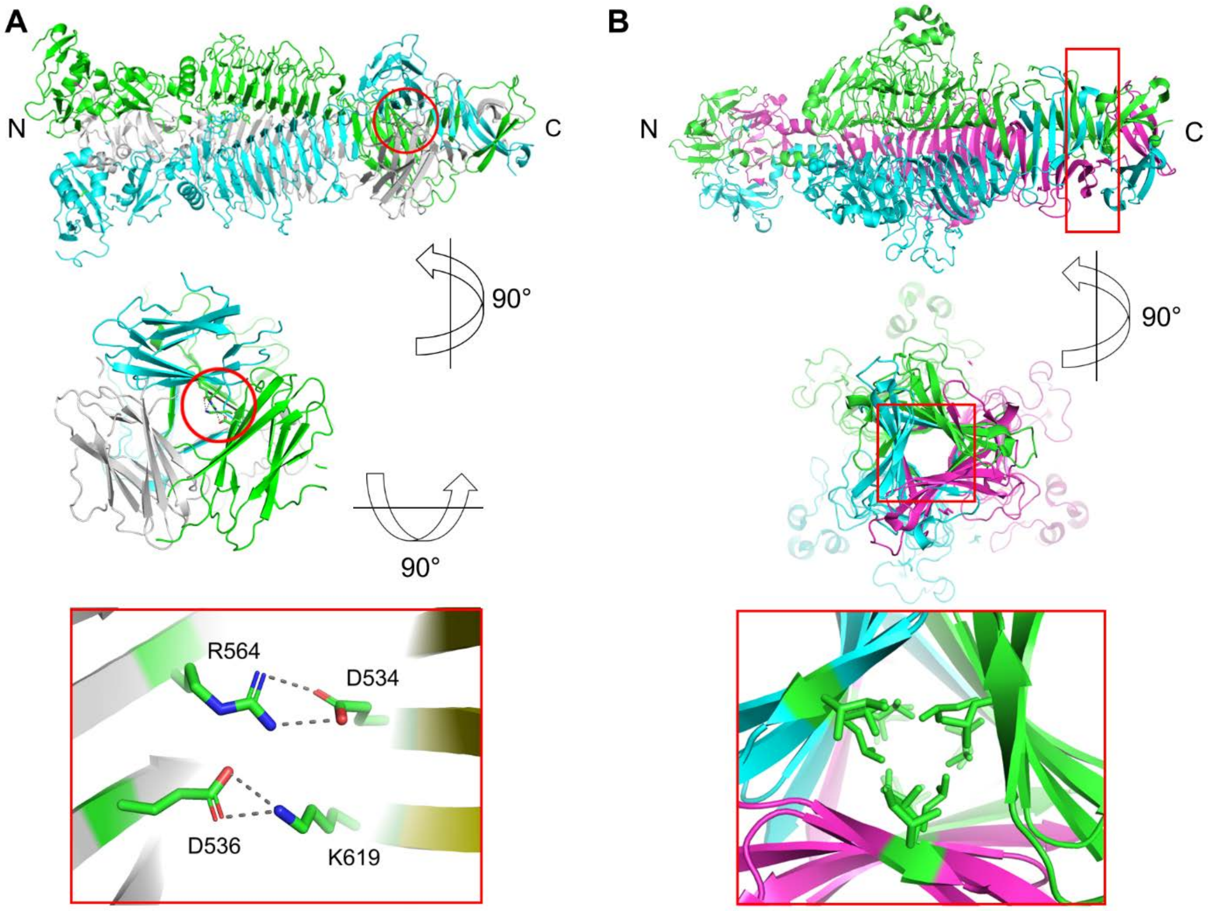


| Protein / residues | Interface area / Å$^2$ | Calculated interface energy / kcal mol$^{-1}$ |
|---|---|---|
| WceF / 34 - 736 | 5140 | -40.5 |
| WceF / 232 - 736 | 4844 | -17.1 |
| P22 / 113 - 666 | 4607 | -35 |

**Figure S9: Details of C-terminal trimer interfaces in WceF and P22TSP and interface area analysis.** Stabilization of native trimers of WceF and P22TSP. (A) Salt bridges interconnecting two neighboring C-terminal sandwich domains in the WceF trimer. (B) Hydrophobic core of the β-prism domain in P22TSP.

**Table S1: Conservation of WceF within *Erwinia* and *Pantoea* species**

BLAST search against database of non-redundant protein sequences with WceF from *Pantoea stewartii* (NCBI accession number WP_006119865.1, 736 aa) as query sequence. For an alignment of the sequences in this table see Figure S1.

| Protein/Species | Size / aa | Sequence identity / % | Origin of bacterial isolate | NCBI accession |
|---|---|---|---|---|
| AmsF /*Pantoea ananatis* | 736 | 96 | Sweet corn, ananas, rice, onion | WP_047714099.1 |
| AmsF /*Pantoea allii* | 736 | 95 | Onion | WP_109717354.1 |
| AmsF / *Pantoea agglomerans* | 738 | 81 | ubiquitous | WP_086906097.1 |
| AmsF / *Pantoea vagans* | 738 | 80 | Eukalyptus | WP_050680143.1 |
| AmsF / *Pantoea cypripedii* | 736 | 78 | Orchid | WP_084875998.1 |
| AmsF / *Erwinia mediterraneensis* | 738 | 75 | Human skin | WP_130830746.1 |
| AmsF / *Erwinia gerundensis* | 740 | 71 | *Rosacea* (e.g. apples, pears) | WP_067432085.1 |
| AmsF / *Erwinia tasmaniensis* | 740 | 67 | | WP_012441069.1 |
| AmsF / *Erwinia amylovora* | 743 | 61 | | WP_004162445.1 |
| AmsF / *Erwinia typographi* | 737 | 54 | Gut contents of bark beetles (*Coleoptera*) | WP_034887639.1 |

**Table S2: X-ray crystallographic data collection and refinement statistics**

| | WceF (SeMet - phasing) | WceF (native) |
|---|---|---|
| **Data collection** | | |
| Beamline | BESSY 14.1 | BESSY 14.2 |
| Wavelength (Å) | 0.9798 | 0.9184 |
| Space group | $P2_1$ | $P2_1$ |
| Cell dimensions | | |
| $a$, $b$, $c$ (Å) | 100.7, 97.6, 129.5 | 100.2, 97.2, 128.9 |
| α, β, γ (°) | 90.0, 106.3, 90.0 | 90.0, 106.4, 90.0 |
| Resolution (Å)* | 34.6 – 2.9 (2.98 – 2.90) | 48.60 – 2.55 (2.70 – 2.55) |
| $R_{meas}$* | 23.6 (60.5) | 23.1 (128.1) |
| $< I / \sigma(I) >$* | 9.72 (1.89) | 6.77 (1.02) |
| CC1/2* | 0.987 (0.668) | 0.985 (0.360) |
| Completeness* (%)* | 99.2 (96.7) | 96.4 (81.4) |
| Redundancy | 7.0 | 3.82 |
| **Refinement** | | |
| Resolution (Å) | | 2.55 |
| No. unique reflections | | 74949 |
| $R_{work}$ / $R_{free}$ (%) | | 22.42 / 26.93 |
| No. atoms | | |
| Protein | | 15839 |
| Ligand | | 194 |
| Water | | 617 |
| Mean $B$ factor (Å$^2$) | | 47.4 |
| R.m.s deviations | | |
| Bond lengths (Å) | | 0.015 |
| Bond angles (°) | | 1.65 |
| Mol/AU | | 3 |

**Table S3: WceF structural homolog prediction with HHPred**

The WceF protein sequence (UniProt H3REJ8) was subjected to HHPred structural homolog prediction.[1] Search was performed against PDB_mmCIF70_3_Aug on August 22$^{nd}$, 2019. 25 best hits according to probability are shown.

| No | pdb code | Homolog prediction | Probability | E-value | aa covered | Total length of homolog |
|---|---|---|---|---|---|---|
| 1 | 5W6H_C | Bacteriophage CBA120 tailspike protein 4 | 100.0 | 2,00E-35 | 403 | (697) |
| 2 | 6NW9_B | Bacteriophage CBA120 tailspike protein 3 | 99.9 | 4.1E-28 | 371 | (633) |
| 3 | 2XC1_C | Bacteriophage P22 tailspike O-antigen recognition domain | 99.9 | 1.1E-24 | 528 | (666) |
| 4 | 5W6S_A | Bacteriophage CBA120 tailspike protein 2 | 99.9 | 1.2E-24 | 324 | (680) |
| 5 | 1LKT_F | Bacteriophage P22 tailspike capsid binding domain | 99.9 | 2.7E-25 | 104 | (104) |
| 6 | 6E1R_E | Acinetobacter phage vB_ApiP_P1, tailspike | 99.9 | 1.7E-22 | 338 | (554) |
| 7 | 4OJ5_A | Bacteriophage CBA120 tailspike protein 1 | 99.8 | 1.1E-21 | 462 | (776) |
| 8 | 2VNL_A | Bacteriophage P22 tailspike capsid binding domain | 99.8 | 4.8E-23 | 118 | (151) |
| 9 | 5W5P_A | Acinetobacter phage AM24 tailspike | 99.8 | 7.1E-21 | 313 | (623) |
| 10 | 6EU4_C | Acinetobacter phage vB_AbaP_AS12 tailspike | 99.8 | 1.5E-20 | 290 | (716) |
| 11 | 1RMG_A | *Aspergillus aculeatus* rhamnogalacturonase A | 99.5 | 4.7E-15 | 262 | (422) |
| 12 | 4C2L_A | *Aspergillus tubingensis* endo-xylogalacturonan hydrolase | 99.5 | 2.5E-14 | 278 | (388) |
| 13 | 2VBK_A | Bacteriophage Sf6 tailspike | 99.4 | 5.7E-14 | 325 | (514) |
| 14 | 1CZF_B | *Aspergillus niger* polygalacturonase II | 99.4 | 8.3E-14 | 252 | (362) |
| 15 | 4XOT_A | Bacteriophage HK620 tailspike | 99.4 | 3.8E-14 | 281 | (597) |
| 16 | 2IQ7_A | *Colletotrichum lupine* endopolygalacturonase | 99.4 | 2.5E-13 | 239 | (339) |
| 17 | 3JUR_C | *Thermotoga maritima* Exo-poly-alpha-D-galacturonosidase | 99.4 | 8.1E-13 | 262 | (448) |
| 18 | 4MXN_B | *Parabacteroides merdae* pectate lyase | 99.3 | 2.7E-13 | 219 | (247) |
| 19 | 1H80_A | *Alteromonas sp.* ATCC43554 iota-carragenase | 99.3 | 4.1E-13 | 233 | (464) |
| 20 | 2UVF_A | *Yersinia enterolytica* exopolygalacturonase | 99.3 | 2.6E-12 | 324 | (608) |
| 21 | 5OLP_A | *Bacteroides thetaiotaomicron* galacturonidase | 99.3 | 3.2E-12 | 300 | (452) |
| 22 | 4RU5_A | Pseudomonas phage phi297 tailspike | 99.3 | 6.2E-12 | 290 | (605) |
| 23 | 1IB4_B | *Aspergillus aculeatus* polygalacturonase | 99.3 | 1.4E-12 | 253 | (339) |
| 24 | 4RU4_F | Pseudomonas phage LKA1 tailspike | 99.3 | 1.3E-11 | 386 | (602) |
| 25 | 1BHE_A | *Chaetomium thermophilum* beta-1,3-glucanase | 99.2 | 9.9E-12 | 295 | (376) |

1. Zimmermann, L., Stephens, A., Nam, S. Z., Rau, D., Kubler, J., Lozajic, M., Gabler, F., Soding, J., Lupas, A. N., and Alva, V. (2018) A Completely Reimplemented MPI Bioinformatics Toolkit with a New HHpred Server at its Core, *J. Mol. Biol. 430*, 2237-2243.
